# Impact of macular fluid volume fluctuations on visual acuity during anti-VEGF therapy in eyes with nAMD

**DOI:** 10.1038/s41433-020-01354-4

**Published:** 2021-01-07

**Authors:** Usha Chakravarthy, Moshe Havilio, Annie Syntosi, Natasha Pillai, Emily Wilkes, Gidi Benyamini, Catherine Best, Alexandros Sagkriotis

**Affiliations:** 1 Centre for Experimental Medicine, Institute of Clinical Science, Queen’s University Belfast, Belfast, UK; 2 Notal Vision Ltd, Tel Aviv, Israel; 3 Novartis Pharma AG, Basel, Switzerland; 4 IQVIA, Basel, Switzerland; 5 IQVIA, London, UK

**Keywords:** Prognostic markers, Prognostic markers, Macular degeneration, Outcomes research

## Abstract

**Objectives:**

To study the effect of repeated retinal thickness fluctuations during the anti-VEGF therapy maintenance phase in neovascular age-related macular degeneration (nAMD).

**Methods:**

Data were extracted from electronic medical records of 381 nAMD patients, aged ≥50 years; baseline VA ≥33 and ≤73 letters; ≥24 months’ follow-up and ≥2 optical coherence tomography (OCT) measurements. OCT scans were analysed using an artificial intelligence algorithm that quantified the volumes of intraretinal fluid (IRF), subretinal fluid (SRF), pigment epithelial detachments (PED) and central subfield thickness (CSFT). IRF, SRF and PED were summed to obtain total fluid (TF). The standard deviation (SD) of IRF, SRF, PED, CSFT and TF was computed and categorised into quartiles (SD-Q). Relationships between SD-Qs for each OCT feature and VA change was tested using generalised estimating equations and linear regression.

**Results:**

By Month 24, compared to SD-Q1, eyes in SD-Q2, SD-Q3, and SD-Q4 for IRF, SRF, PED, CSFT and TF showed greater VA losses. Eyes in SD-Q4 of TF were 9.4 letters worse compared to eyes in Q1 (95% Confidence Interval: −12.9 to −6.0). The frequency of clinic visits with IRF and SRF present on OCT scans by quartiles of CSFT was lower in eyes with least fluctuation (Q1) compared to eyes with the most fluid fluctuation (Q4) (median [IQR] IRF: 0.3 [0.0–0.7] versus 0.8 [0.5–1.0]; SRF: 0.0 [0.0–0.5] versus 0.6 [0.3–1.0]).

**Conclusions:**

Greater fluctuations in retinal fluid volumes during the maintenance phase of anti-VEGF treatment in nAMD is associated with worse VA by 2 years.

## Introduction

Neovascular age-related macular degeneration (nAMD), a late stage manifestation of age-related macular degeneration, is characterised by new blood vessel formation in the macula of the eye. These new vessel complexes leak fluid and blood, distort the retinal architecture and result in sudden onset vision loss which, if left untreated, can progress rapidly to severe and permanent vision impairment [1]. Anti-vascular endothelial growth factor (anti-VEGF) therapies have become the standard of care for nAMD, resulting in improvement in vision and prevention of progression to severe vision loss in over 90% of treated patients over a 2 year period [2, 3]. The anti-VEGF treatment period is typically composed of monthly injections for a period of 3 months (i.e., the loading phase) followed by a maintenance phase which continues ad infinitum. Re-treatments are administered either at fixed dosing intervals or as flexible pro re nata or treat-and-extend regimens [4].

Despite reductions in the incidence of blindness and severe vision loss since the introduction of anti-VEGF agents, long term follow up studies show that a proportion of eyes continue to lose visual acuity (VA) [5, 6]. Comparisons of outcomes in countries employing different strategies for monitoring and re-treatment suggest that suboptimal treatment frequency and an incomplete loading phase are factors that contribute to vision loss [7]. It is recognised that sub-optimal dosing frequency causes recurrence of exudation, a marker of which are fluctuations in retinal thickness [8–10]. However the central retinal subfield thickness (CSFT) fluctuations which occur when prolonging re-treatment intervals have not been shown to affect the visual acuity outcomes 2 years after initiation of treatment in comparative effectiveness trials. Nevertheless, these studies looked at average outcomes and did not take into consideration the severity of the fluctuation and nor did they test the trajectory of vision loss by severity of fluctuation. The standard deviation (SD) of an OCT metric, measured at multiple visits can be considered a marker for repeated cycles of retinal thinning and thickening, representing the severity of thickness fluctuations over time. In the present study we examined the relationship between the SD of a number of measures of OCT reflecting lesion activity and visual acuity during the maintenance phase of anti-VEGF therapy and at the end of 2 years of treatment.

## Methods

This study was designed, conducted, and reported in accordance with the guidelines for Good Pharmacoepidemiology Practices of the International Society for Pharmacoepidemiology, Strengthening the Reporting of Observational Studies in Epidemiology, and the Declaration of Helsinki [11–13].

Clinical Data: This was a retrospective analysis of data obtained from a single site in the United Kingdom, with clinical information that had been captured in a standardised manner on the Medisoft electronic medical record (EMR) platform and integrated with OCT metrics obtained through artificial intelligence (AI) analysis of the corresponding image repository.

Following approval from the data guardian of the clinical site, pseudo-anonymized data that spanned the period April 2010 and October 2019 were exported by the data controller without any patient identifiers. The exported data were scrutinised by an independent statistical team at the *Data Science Hub, Real World Solutions (IQVIA, London, UK and Basel, Switzerland)* and cases were extracted if they fulfilled the following criteria: age ≥50 years, treated with any licensed anti-VEGF agent, diagnosis of nAMD only, baseline VA between ≥33 and ≤73 Early Treatment Diabetic Retinopathy Study (ETDRS) letters, ≥24 months of follow-up, and ≥2 OCT scans captured during the maintenance phase. Data from both eyes were included if the condition was bilateral.

Image data: The image repository of OCT scans, captured on the Spectralis device, (Heidelberg Engineering, Heidelberg, Germany) and corresponding to the selected case ID’s were similarly anonymized and exported as standard Audio Video Interleave files by the data controller of the hospital for AI analysis.

**Fig. 1:**
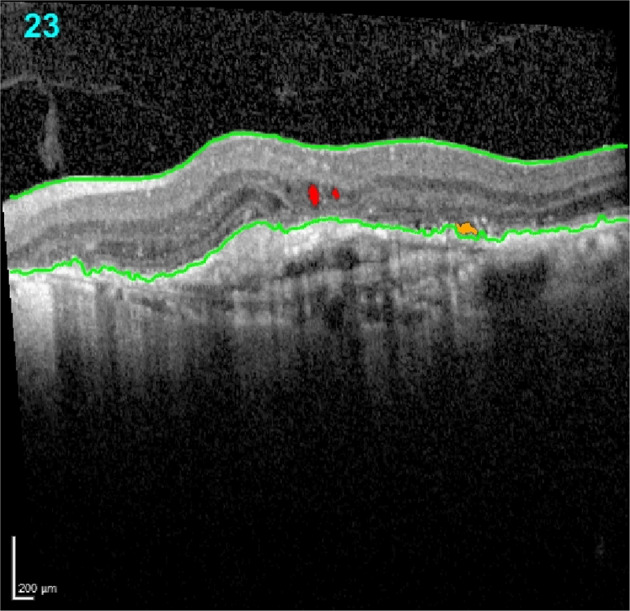
Representative B scan segmented using the NOA algorithm. The ILM and the hyperreflective RPE band are outlined in green. Two foci of intraretinal fluid are shown delineated in red. Subretinal fluid lying between the RPE band and the outer limits of the photoreceptor layer is seen in orange. ILM, inner limiting membrane; RPE, retinal pigment epithelium.

Image analysis: The anonymized cube scans were analysed using an AI software algorithm (Notal OCT Analyzer [NOA], Notal Vision Ltd., Tel Aviv, Israel) that segments B scans in an automated manner. The AI algorithm, its application in nAMD and its diagnostic accuracy has been previously described and validated on both Cirrus (Carl Zeiss Meditec, Dublin, CA, USA) [14] and Spectralis systems [14, 15]. The NOA software was used to automatically detect and segment OCT markers of lesion activity (Fig. 1). Automated volumetrics were obtained for intraretinal fluid (IRF), subretinal fluid (SRF), pigment epithelial detachment (PED) and the CSFT based on the cube scan of the OCT for each visit. A derived measure was the total fluid (TF) volume, which was defined as the sum of IRF, SRF and PED.

To validate the NOA for the present analysis, a subset of 288 OCT cube scans from 47 eyes representing 5.5% of 5251 OCT images analysed in the study was used. Validation included determination of the presence or absence of IRF and/or SRF by a retina expert and served as the reference standard.

### Statistical analysis

Data management: Owing to the real-world nature of the data, VA measurements from the clinical dataset were not always made at the exact time points specified in this study. A 30-day time window was therefore applied to assign VA measurements to visits at Months 0, 3, 6 and 9, and a 60-day time window was applied to Months 12, 18 and 24. A 3-week time window was used to match OCT scans to VA measurements at the specified time points. Any VA not recorded as ETDRS letter scores were converted from logarithm of Minimum Angle of Resolution to ETDRS letter scores according to established guidelines [16].

For each patient, the within-patient SD for IRF, SRF, PED, CSFT and TF from all analysed OCT scans from all available visits between Months 3–24 were calculated. The SD is a proxy measure for the severity of volume fluctuations. The SD distribution for each OCT marker of lesion activity as well as TF was examined, and quartiles were generated with quartile 1 reflecting the least variability and quartile 4 the greatest variability.

Initial data extraction and generation of descriptive characteristics were performed on SAS 9.2 (SAS Institute Inc., Cary, NC, USA). All subsequent analyses were performed on R 3.6.1. Descriptive statistics were tabulated for demographic and clinical characteristics. Continuous variables were summarised as number of observations, means and their SD. Spearman’s rank correlation coefficients were generated for the OCT markers of lesion activity. Multivariate generalised estimating equations (GEEs) were built to model the association between SD of each OCT feature (IRF, SRF, PED, CSFT and TF) and change in VA from Month 3 to Month 24, adjusting for age, baseline VA and lesion size, including fixed and time-varying measurements and the use of data from two eyes of any one patient. The dependent variable in each case was the change in VA from Month 3 to each study time point (i.e., Months 6–24). Independent variables were age at baseline, VA at baseline and at Month 3, completion of a loading phase (≥3 injections in the first 90 days; no/yes), the SD quartile of the volume of the OCT feature of interest, the injection rate up to the analysed time point (i.e., Months 6–24), the study time point (as a categorical variable) and an interaction term between SD quartiles and time that allowed the effect of quartile to vary at each time point.

To demonstrate the adjusted mean VA change from Month 3, estimated marginal means (least squares means) at each time point (i.e., Months 6–24) were calculated for each SD quartile. Adjusted mean differences were calculated to compare each SD quartile (Q2–Q4) to the lowest (reference) quartile (i.e., Q1) at Month 24.

Data from a cohort of patients with ≥24 months follow-up, but with missing VA at Month 24 (*n* = 413) were not included in the main analysis but were tested in a sensitivity analysis where missing VA at Month 24 was imputed using linear interpolation.

In the linear interpolation model all independent variables from the multivariate GEE model were fitted with exception of ‘time’ and ‘time-by-SD quartile interaction’. Mean changes in VA from baseline to Month 24 and from Month 3 to Month 24 were calculated using this imputed VA.

The proportion of visits per eye with IRF and SRF presence by SD quartile of CSFT was also examined.

## Results

### Baseline characteristics

The number of eligible eyes by pre-specified eligibility criteria and reasons for exclusion are shown in Supplementary Fig. S1. On application of all eligibility criteria, data from 403 eyes from 381 cases was available and constituted the main cohort. An additional set of 413 eyes from 399 patients who fulfilled all selection criteria except for a lack of matched VA and OCT at Month 24 was used in the sensitivity analyses. Demographic and baseline characteristics of the main cohort and that used for the sensitivity analysis are shown in Table 1. The mean age of cases included in the main cohort was 77.8 (6.8) years and was similar to the 399 cases who contributed 413 eyes to the sensitivity analysis (Table 1). The number of eyes that have been analysed at each time point is presented in Supplementary Table S1 and the number of visits and number of eyes included for calculation of SD quartiles for each OCT parameter is presented in Supplementary Fig. S2.

**Table 1:** Demographic and clinical characteristics at baseline.

Characteristics	OCT cohort (*N* = 403 eyes from 381 patients)	Cohort with limited data at Month 24 (*N* = 413 eyes from 399 patients)
Age at index (patient, years)		
Mean (SD)	77.8 (6.8)	79.6 (6.3)
Gender (patient; *n*, %)		
Male	141 (37.0%)	142 (35.6%)
Female	240 (63.0%)	257 (64.4%)
Treatment laterality (patient; *n*, %)		
Unilateral	359 (94.2%)	385 (96.5%)
Bilateral	22 (5.8%)	14 (3.5%)
VA study eye (ETDRS letters)*		
Mean (SD)	58.2 (10.4)	54.6 (11.3)
IRF volume (mm^3^; *n*, %)		
Mean (SD)	0.14 (0.26)	0.14 (0.25)
SRF volume (mm^3^; *n*, %)		
Mean (SD)	0.18 (0.28)	0.16 (0.28)
PED volume (mm^3^; *n*, %)		
Mean (SD)	0.73 (0.96)	0.74 (0.82)
Total fluid volume (mm^3^; *n*, %)**		
Mean (SD)	1.05 (1.18)	1.04 (0.99)
CSFT (microns)		
Mean (SD)	350.1 (109.7)	337.1 (109.3)

*VA at baseline or in the 30-day pre-baseline period. **Total fluid volume is defined as the sum of IRF, SRF and PED fluid volumes.

*CSFT* central subfield thickness, *ETDRS* Early Treatment Diabetic Retinopathy Study, *IRF* intraretinal fluid, *OCT* optical coherence tomography, *PED* pigment epithelial detachment, *SD* standard deviation, *SRF* subretinal fluid

### Relationship between SD quartiles of OCT markers of lesion activity and associations with VA outcomes

The sensitivity and specificity of the NOA for identification of IRF and SRF were 95% (95% Confidence Interval [CI]: 91–98%) and 94% (95% CI: 87–98%), respectively.

The mean volume for each OCT marker of lesion activity IRF, SRF, PED, CSFT and TF at baseline and the mean and SD for each parameter across all visits from Month 3 to Month 24 are shown in Table 2. The correlation plot (Supplementary Fig. S3) shows that CSFT correlated strongly with IRF and moderately with SRF. Variations in mean CSFT over time for the four CSFT SD quartiles are shown in Supplementary Fig. S4.

**Table 2:** Mean volumes of OCT markers of lesion activity at baseline and during the maintenance phase (Month 3–24) of anti-VEGF treatment.

OCT marker of lesion activity	Baseline visit	Month 3 to Month 24 visits	Maintenance phase			
			SD-Q1 (SD range)	SD-Q2 (SD range)	SD-Q3 (SD range)	SD-Q4 (SD range)
IRF (mm^3^; mean [SD])	0.14 (0.26)	0.03 (0.11)	0.000–0.000	0.001–0.006	0.007–0.028	0.029–1.01
SRF (mm^3^; mean [SD])	0.18 (0.28)	0.04 (0.12)	0.000–0.000	0.001–0.008	0.009–0.043	0.044–0.699
PED (mm^3^; mean [SD])	0.73 (0.96)	0.55 (0.49)	0.000–0.036	0.037–0.068	0.069–0.123	0.124–2.79
CSFT (microns; mean [SD])	350.1 (109.7)	239.3 (64.9)	0.000–11.6	11.7–24.2	24.3–44.1	44.2–172
Total fluid SRF + IRF + PED (mm^3^; mean [SD])	1.05 (1.18)	0.62 (0.56)	0.000–0.047	0.048–0.093	0.094–0.187	0.188–3.09

The SD ranges for the four SD quartiles of each OCT marker of lesion activity (IRF, SRF, PED, CSFT and total fluid) are shown in columns 4–7. *CSFT* central subfield thickness, *IRF* intraretinal fluid, *OCT* optical coherence tomography, *PED* pigment epithelial detachment, *SD* standard deviation, *SD-Q* SD-quartile, *SRF* subretinal fluid, *VEGF* vascular endothelial growth factor

The distribution of the number of visits from which volumes of IRF, SRF, PED, CSFT and TF was available and used to derive SD quartiles are shown in Supplementary Fig. S2. On cross tabulation approximately one third of eyes were placed in equivalent categories when classified by IRF/SRF, IRF/PED or SRF/PED SD quartiles (Supplementary Table S2, available at https://ophthalmologyretina.org/). Marginal differences in baseline VA were observed across quartiles but varied by the OCT marker of lesion activity. Lesion size differed significantly across quartiles (Supplementary Tables S3–S7, available at https://ophthalmologyretina.org/).

**Fig. 2:**
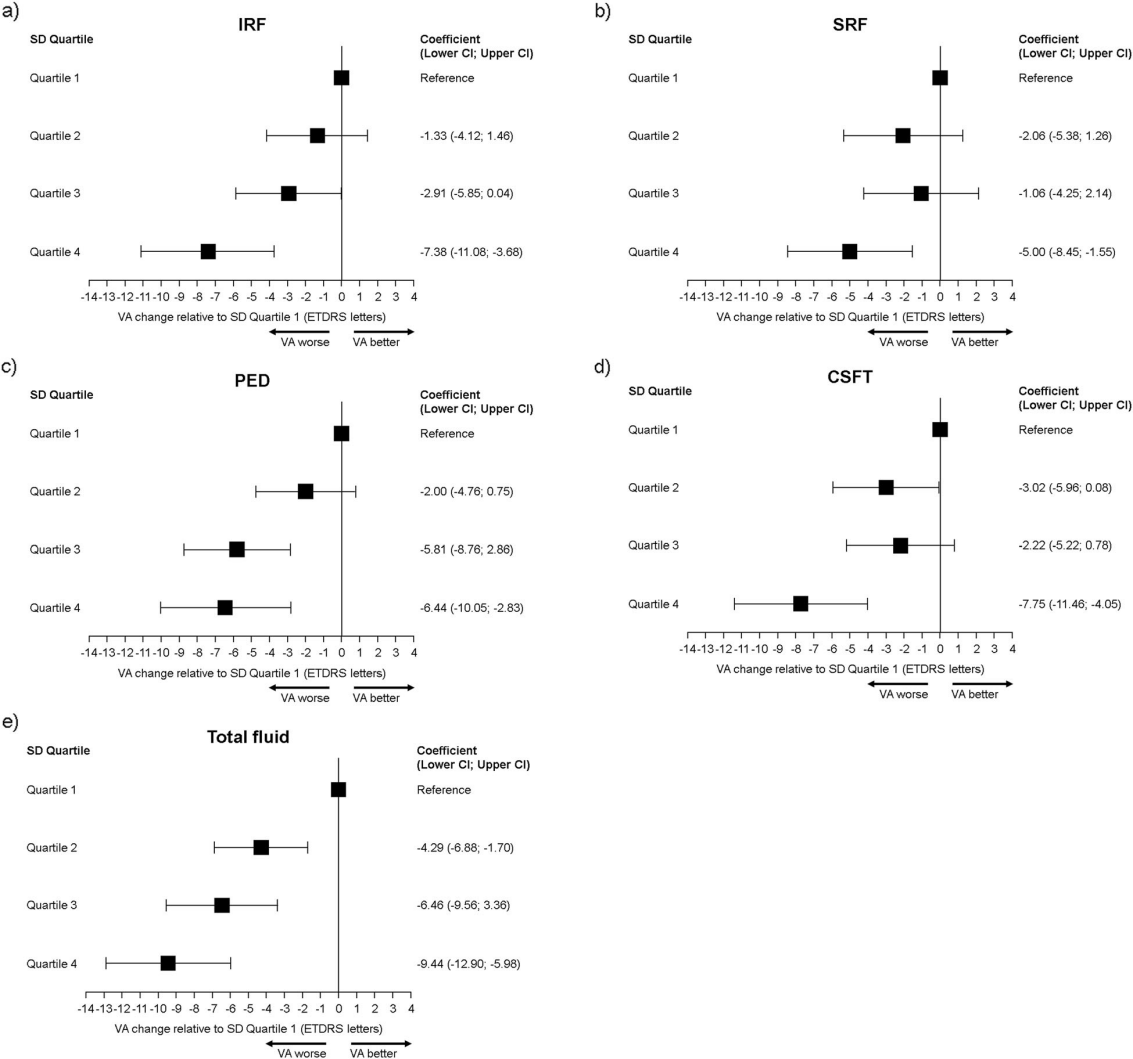
GEE models of forest plots of adjusted mean change in VA from Month 3 to Month 24 by SD quartile relative to uartile 1 for each OCT marker of lesion activity. VA changes for Q1 to Q4 for the OCT markers IRF (**a**), SRF (**b**), PED (**c**), CSFT (**d**), and total fluid (sum of IRF + SRF + PED) (**e**). The standard deviation for each OCT marker of lesion activity was calculated from all available visits. Point estimates for VA change with 95% CI are shown at Month 24. CI, confidence interval; CSFT, central subfield thickness; GEE, generalised estimating equation; IRF, intraretinal fluid; OCT, optical coherence tomography; PED; pigment epithelial detachment; SD, standard deviation; SRF, subretinal fluid; VA, visual acuity.

The relationship between changes in VA from completion of the loading phase to Month 24 for each OCT marker of lesion activity by quartile is shown in forest plots in Fig. 2. The point estimates of the difference in VA at Month 24 increased by increasing quartile for IRF and TF. For SRF, the point estimates and the 95% confidence interval (CI) limits for the difference in VA change at Month 24 in Q2 and Q3 were similar to Q1 but Q4 showed a significantly greater loss of 5 letters of VA. The models for PED and CSFT also showed worsening point estimates by quartile for the VA difference at Month 24. In all models the lower limit of the CIs in Q4 for VA change did not include zero. The forest plots (Fig. 2) show that the most marked difference in VA was seen for TF (IRF + SRF + PED) with eyes in Q4 experiencing a loss of −9.4 letters (95% CI −12.9, −6.0) compared to Q1.

Differences in the trajectory of VA change by quartile was seen for the 5 OCT markers of lesion activity (Supplementary Fig. S5). For IRF the trajectory of VA change showed some overlap of Q1 and Q3 during the first year but with divergence of the 4 categories beyond Month 12 (Supplementary Fig. S5A). For SRF there was overlap of the trajectory of VA change in Q2 and Q3 during the 24 month period (Supplementary Fig. S5B). For CSFT Q2 showed a steeper decline in VA compared to Q3 but Q4 showed the steepest drop in VA (Supplementary Fig. S5D). The separation of the trajectory of VA change by quartile was best seen with respect to PED where divergence between categories was evident by Month 9 (Supplementary Fig. S5C). The VA trajectory for TF showed that Q1 had the best outcome with VA continuing to rise between Month 3 and Month 24 (Supplementary Fig. S5E). All other quartiles showed steady reductions in VA with the worst outcome in Q4.

Supplementary Fig. S6 shows the proportion of visits per eye at which IRF and SRF were present when classified by CSFT SD quartile. The proportion of visits with IRF present was lowest in CSFT SD Q1 and highest in Q4. Visits with SRF present was also low in Q1 but the relationship of increasing frequency was not a feature in Q4 which showed a lower frequency of SRF free visits compared to Q3.

### Sensitivity analysis

Patients in the limited data cohort had lower baseline VA (54.6 [11.3] ETDRS letters), gained fewer letters from baseline to Month 3 (3.0 [9.4]) and received fewer injections (5.4 [4.5]) compared with the 403 eyes in the main cohort. OCT metrics and completion of loading phase were similar between the two cohorts (Table 1 and Supplementary Table S8).

## Discussion

This study used AI guided quantification of OCT markers of lesion activity by location within the retina and tested associations with the severity of thickness fluctuations over time. Our findings support the view that visual acuity is significantly worse by around two lines after 2 years of anti-VEGF treatment in eyes that experience the most fluctuations in retinal thickness. Notably we have shown that fluctuations attributable to IRF had the greatest impact on VA. Eyes in CSFT-SD Q3 and Q4 were more likely to have a higher frequency of visits at which IRF or SRF were present.

This method of using the SD of an OCT metric captured at multiple visits was first used in a meta-analysis of the CATT and IVAN trials. In the combined dataset the authors used the SD of the foveal centre point thickness (FCPT) from multiple visits spanning the 2 year trial period and showed worse VA outcomes in eyes that experienced most fluctuations in FCPT compared to those with least fluctuations [17]. Because the majority of participants in the CATT and IVAN trials had been imaged on time domain OCT instruments, their data was restricted to a point measurement of retinal thickness at the fovea. By contrast, the image repository in the present study consists of spectral domain OCT, which provides high resolution cube scans of the central macula, and our AI algorithm analysed the scans from all available clinic visits over 24 months. This allowed us to obtain volume measurements for each morphological marker of lesion activity and separately assign these components to specific tissue layers.

After 2 years of anti-VEGF treatment, eyes in the higher SD quartiles of all OCT markers of lesion activity were significantly worse compared to the reference Q1. Our findings support those of the CATT and IVAN meta-analysis in that fluctuations in retinal thickness due to intermittent cycles of quiescence and lesion activity had an adverse effect on BCVA at Month 24 [17].

Because we were able to use AI analytics to segment the macular rasters, we obtained information on the localisation of fluid to specific retinal tissue compartments and its effect on visual function. Specifically, the almost monotonic worsening of VA loss by quartile was most pronounced for IRF and least pronounced for SRF. In a dataset of patients with nAMD managed with anti-VEGF therapy, Schmidt Erfurth et al. have shown that the horizontal extent of IRF measured using AI analytics at baseline and at months 1–3 was the best predictor of BCVA at month 12. They comment that IRF was the most relevant biomarker while SRF and PED were ranked low [18]. IRF is typically associated with Type III CNV [19] and our findings suggest that recurrence of disease activity in this phenotype likely results in greater neuro-retinal tissue compartment volume fluctuations. Interestingly, the worst VA outcome with a loss of nearly two lines at Month 24 was in Q4 of TF. The TF is a marker for fluid in the retina, subretinal and sub-pigment epithelial spaces. It was notable that in Q4 of TF, VA was almost two lines worse compared to Q1. Our findings therefore suggest that large fluctuations in any of the tissue compartments in which fluid builds up has an unfavourable effect on visual outcome.

The proportion of visits at which IRF was present when binarized to 0 or >0 mm [3] from the AI outputs was least in the lowest quartiles of CSFT fluctuation rising to more than three quarters of visits in Q4. Interestingly the proportion of visits with SRF increased with increasing quartile but dropped in Q4, suggesting that SRF was not a major contributor to larger or more persistent fluctuations in retinal thickness. It was notable that SD-Q3 and 4 derived from PED volumes were associated with significantly worse VA (Fig. 2). Our findings therefore imply that unless large fluctuations in PED volume are observed, shallow unchanging PED’s are more benign. In this context our data support the view that PED’s may be beneficial when not associated with other markers of lesion activity [20–22].

In the FLUID study, a fluid intolerant treatment regimen was compared with a relaxed regimen, and no statistically difference in BCVA was reported at 24 months [18]. Although FLUID did not evaluate fluctuations in retinal volume, it was interesting that only SRF was tolerated, but not IRF. Our data show that fluctuations in IRF rather than SRF is the driver for the worst functional outcome. Therefore, our data while not directly comparable to the FLUID study is indeed partially supported by their findings.

Our study has several limitations. Only 22% (*n* = 403) of eyes from the initial cohort of 1864 eyes contributed to the main analysis at Month 24. One reason for the high proportion of missing information was because we limited the extraction of data for analysis to that which was entered in the drop down fields of the EMR and this was only completed in a minority of patients. We did not use manual or AI techniques to extract information that was entered into free text fields of the EMR. Other reasons for exclusions were missing matched VA and OCT at Month 24 which was our pre-specified primary outcome point. However, we undertook a sensitivity analysis of the 413 eyes that were excluded due to absence of matched VA with OCT at Month 24. In this cohort, baseline VA and OCT characteristics were similar to the main cohort but we did observe less VA gains at the end of the 3-month loading phase. A small proportion of included eyes were from patients with bilateral disease, however we accounted for potential co-linearity of outcomes in the two eyes of an individual by the use of GEEs. In addition, the visit numbers used to calculate the SD of fluctuations varied extensively across the population due to the real-world nature of this study. Another limitation of our study is the size of the patient sample which was moderate, nonetheless the observed demographics and clinical characteristics in this study were comparable to previous real-world nAMD studies [22, 23].

Strengths of this study include determination of the volumes of markers of lesion activity within the macula over time and the calculation of a measure of the severity of fluctuations in these volumes. We localised fluid to distinct macular tissue compartments and we used AI to analyse all OCT B scans of the macular raster for volume quantifications rather than simple two dimensional metrics. By examining the change from Months 3 to 24, we excluded the impact of macular volume changes during the loading phase.

In conclusion, our study has characterised the volume changes over time in multiple tissue compartments of the macula and demonstrated an adverse relationship between repeated cycles of lesion activity and quiescence and VA outcomes. Measuring changes in the various retinal tissue compartments is hugely time consuming if performed manually. Our study has highlighted the detrimental effects on VA from macular volume fluctuations. This knowledge is likely to trigger the use of AI analytics which are already available from multiple sources [17, 18]. We therefore expect such algorithms to become part of routine clinical practice over time and will be particularly applicable when patients have had treatment for 6 months or more. Such algorithms have the potential to identify subsets of patients who may need more intensive management.

Our data emphasise the importance of optimal management during the maintenance phase of anti-VEGF treatment algorithms and indicate that stringent control of retinal volume fluctuations will help in preventing the decline of VA over time. We also contend that the use of drugs with greater durability and slow release formulations are likely to yield better visual outcomes along with the reduction in treatment burden.

### Summary

#### What was known before

- Long-term follow-up studies show that a proportion of nAMD eyes continue to lose visual acuity despite anti-VEGF treatment.
- This may be due to sub-optimal dosing frequency causing recurrence of retinal fluid accumulation, a marker of which are fluctuations in retinal thickness.

#### What this study adds

- We used a validated artificial intelligence supported method to examine the relationship between fluctuations in several measures of OCT markers of lesion activity and visual acuity during the maintenance phase of anti-VEGF therapy and at the end of 2 years of treatment.
- Our data shows that greater fluctuations in retinal fluid volumes during the maintenance phase of anti-VEGF treatment in nAMD is associated with worse VA after 2 years.
- nAMD eyes with the most retinal fluid fluctuation lost 9.4 letters more than eyes with the least fluid fluctuations. Fluctuations in intraretinal fluid had a larger impact on vision than subretinal fluid.

## Supplementary information

Supplementary information
