# Supplementary material for: Impact of macular fluid volume fluctuations on visual acuity during anti-VEGF therapy in eyes with nAMD
Source: Eye (Lond). 2021 Jan 7;35(11):2983–90. doi: 10.1038/s41433-020-01354-4 (PMC8526705; doi:10.1038/s41433-020-01354-4)
Supplement: Supplementary file 1 — Supplementary information [file 41433_2020_1354_MOESM1_ESM.pdf]

Supplementary figures and tables

Supplement Figure 1. Selection criteria (inclusion and exclusion) and definitions in the identification of the study cohort. 3

Supplement Figure 2. Number of visits and number of eyes included for calculation of SD quartiles for each OCT parameter. 4

- **a) IRF** 4
- **b) SRF** 5
- **c) PED** 5
- **d) CSFT** 6
- **e) Total fluid (IRF+SRF+PED)** 6

Supplement Figure 3. Correlations between the OCT markers of lesion activity IRF, SRF, PED, CSFT and Total Fluid (IRF+SRF+PED). 7

Supplement Figure 4. Mean changes in CSFT over time for eyes in the four CSFT SD quartiles. 8

Supplement Figure 5. Trajectory of adjusted estimated marginal mean (LS mean) change in VA from month 3 to month 6, 9, 12, 18 and 24 by SD quartile from GEE models. 9

- **a) IRF** 9
- **b) SRF** 10
- **c) PED** 10
- **d) CSFT** 11
- **e) Total fluid (IRF+SRF+PED)** 11

Supplement Figure 6. Box and whisker plots showing distribution of the proportion of clinic visits per eye with presence of IRF and SRF during the maintenance phase of anti-VEGF therapy (Month 3 to Month 24) stratified by SD quartile of CSFT. 12

Supplement Table 1. Number of eyes available for analysis in SD quartiles 1 to 4 at Months 3–24. 13

Supplement Table 2. Cross tabulation of the distribution of eyes in IRF, SRF and PED SD quartiles. Grey shading indicates eyes classified in equivalent SD quartiles (IRF/SRF: 33.2%; IRF/PED: 36.0%; SRF/PED: 39.0%). 14

Supplement Table 3. Patient and ocular characteristics by IRF SD quartiles in cohorts included in the main and sensitivity analyses. 15

Supplement Table 4. Patient and ocular characteristics by SRF SD quartiles in cohorts included in the main and sensitivity analyses. 16

Supplement Table 5. Patient and ocular characteristics by PED SD quartiles in cohorts included in the main and sensitivity analyses. 17

Supplement Table 6. Patient and ocular characteristics by CSFT SD quartiles in cohorts included in the main and sensitivity analyses. 18

Supplement Table 7. Patient and ocular characteristics by total fluid (IRF+SRF+ PED) SD quartiles in cohorts included in the main and sensitivity analyses. 19

Supplement Table 8. Number of injections and changes in VA over time in eyes without missing OCT metrics (N=403) compared with eyes with missing metrics (N=413). 20

Supplement Figure 1. Selection criteria (inclusion and exclusion) and definitions in the identification of the study cohort.

ETDRS, Early Treatment Diabetic Retinopathy Study; nAMD, neovascular age-related macular degeneration; OCT, optical coherence tomography; VA, visual acuity; VEGF, vascular endothelial growth factor; VMI, vitreomacular interface.

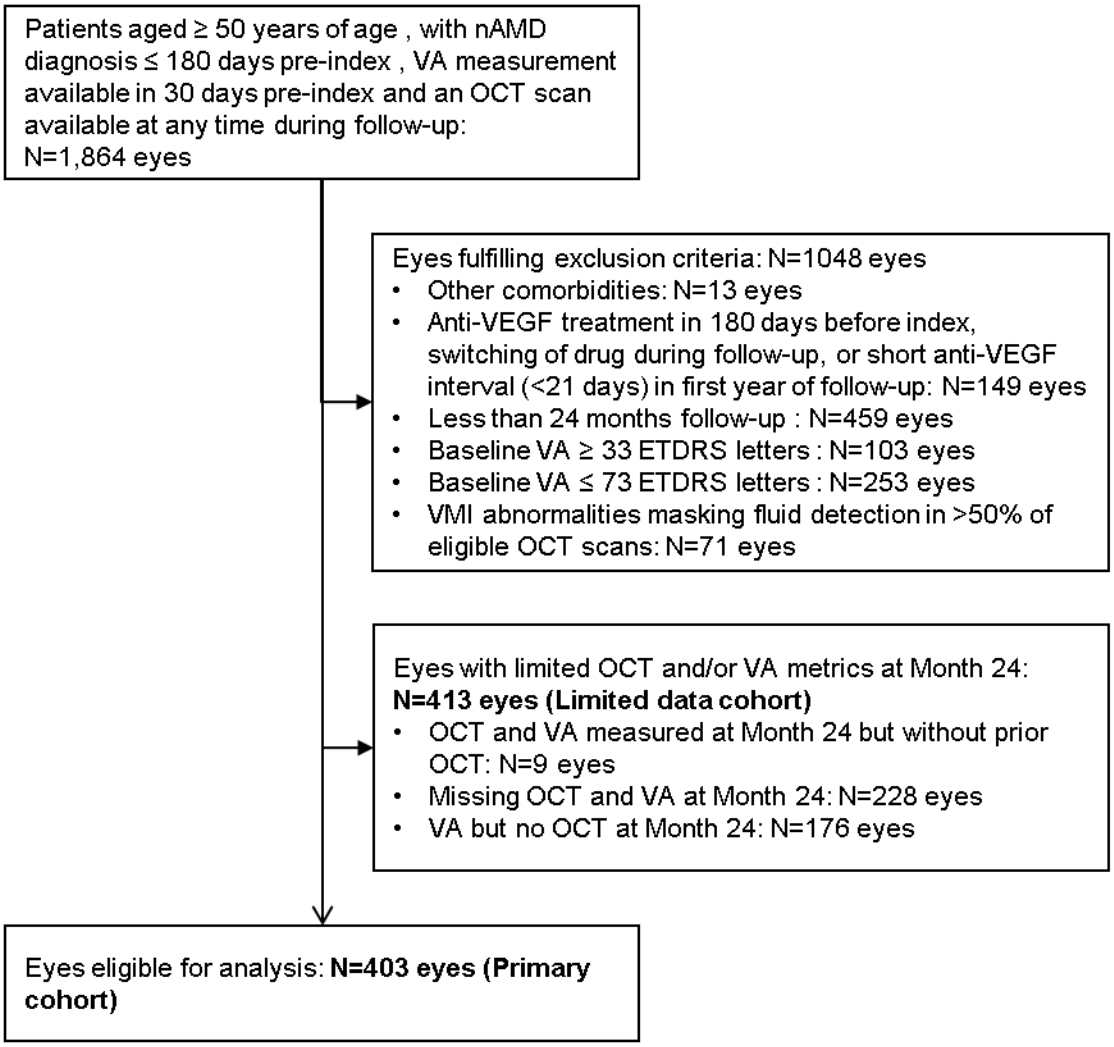

Supplement Figure 2. Number of visits and number of eyes included for calculation of SD quartiles for each OCT parameter.

Panels a–e display bar graphs showing the distribution of the number of visits from which data was available and used in the calculation of SD quartiles for each of the OCT parameters of IRF (a), SRF (b), PED (c), CSFT (d) and TF (IRF+SRF+PED). CSFT, central subfield thickness; IRF, intraretinal fluid; SRF, subretinal fluid; PED, pigment epithelial detachment.

a) IRF

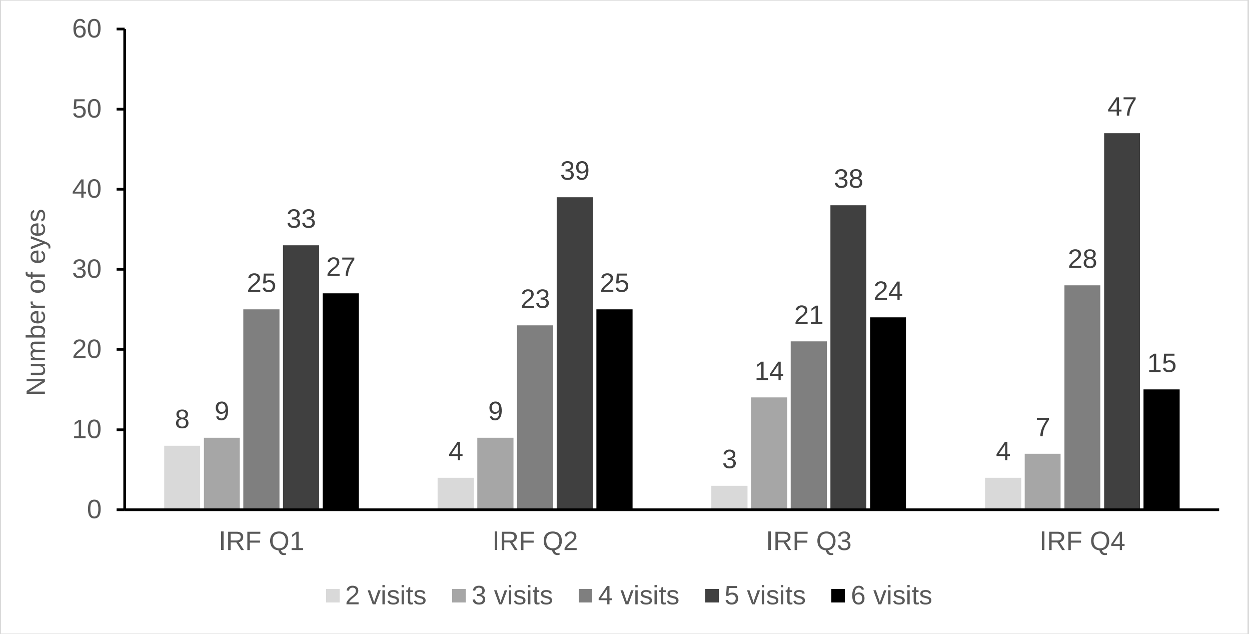

b) SRF

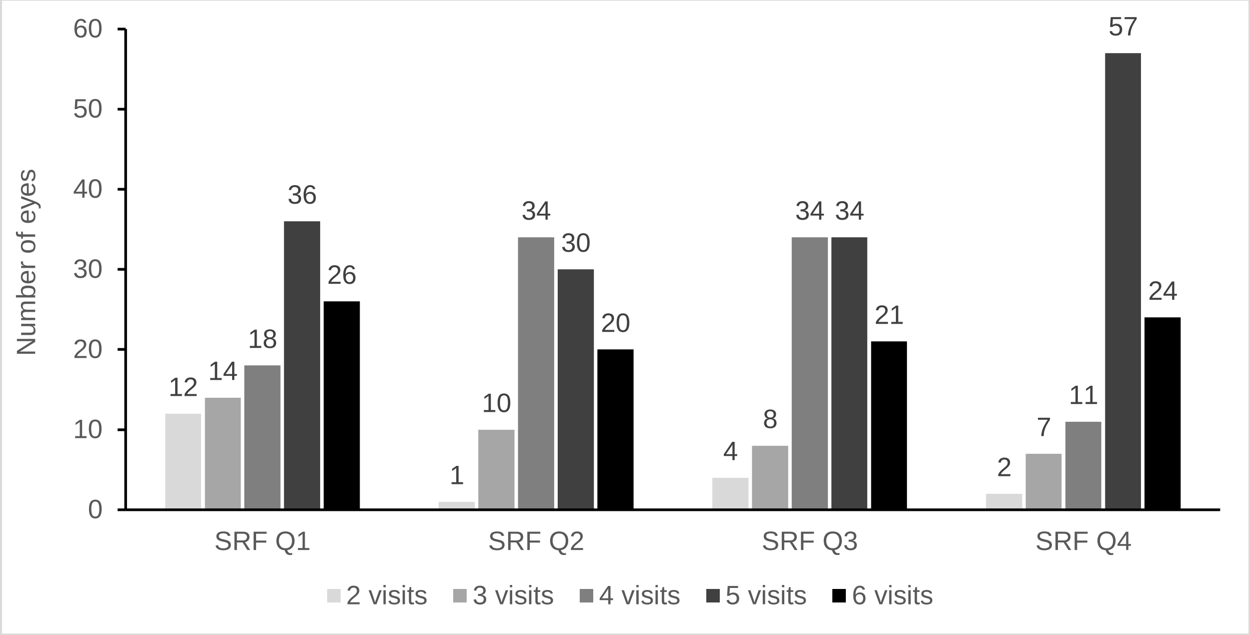

60
50
40
30
20
10
0
Number of eyes
12
14
18
36
26
1
10
34
30
20
4
8
34
34
21
2
7
11
57
24
SRF Q1
SRF Q2
SRF Q3
SRF Q4
2 visits
3 visits
4 visits
5 visits
6 visits

c) PED

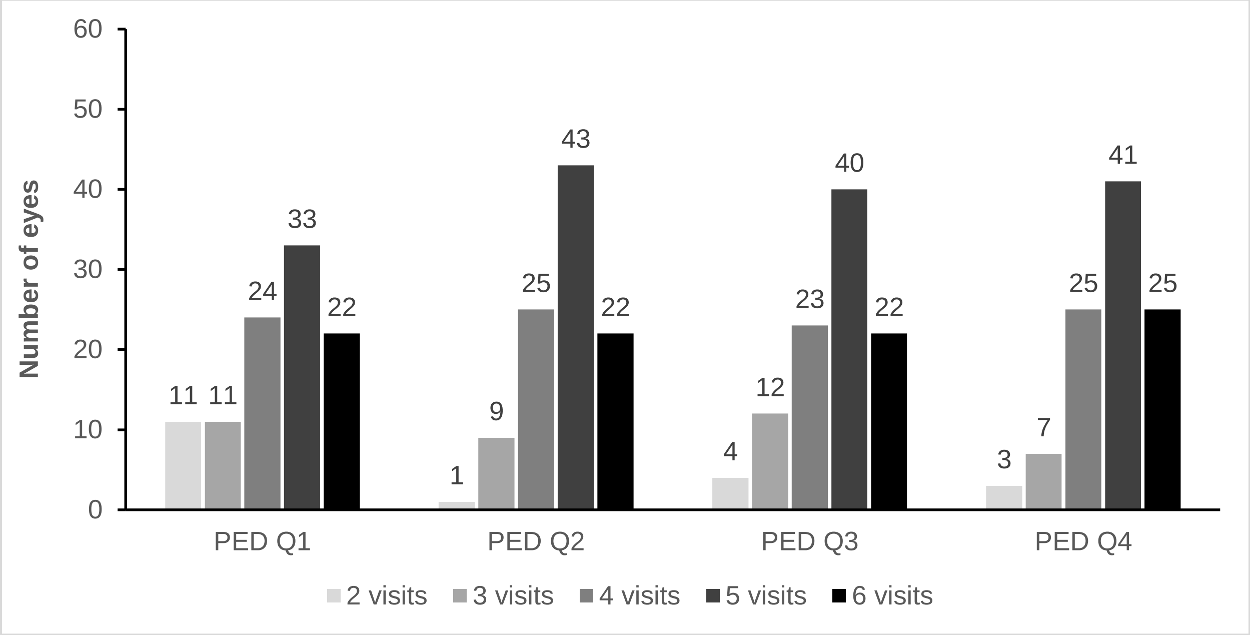

60
50
40
30
20
10
0
Number of eyes
11
11
24
33
22
1
9
25
43
22
4
12
23
40
22
3
7
25
41
25
PED Q1
PED Q2
PED Q3
PED Q4
2 visits
3 visits
4 visits
5 visits
6 visits

d) CSFT

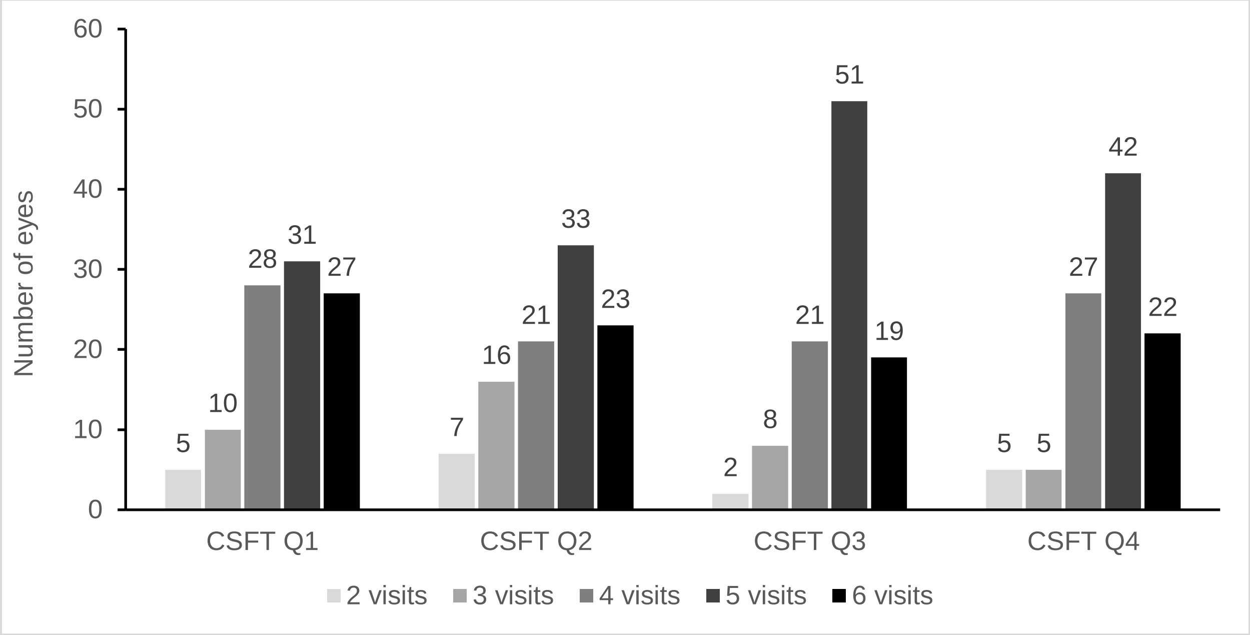

e) Total fluid (IRF+SRF+PED)

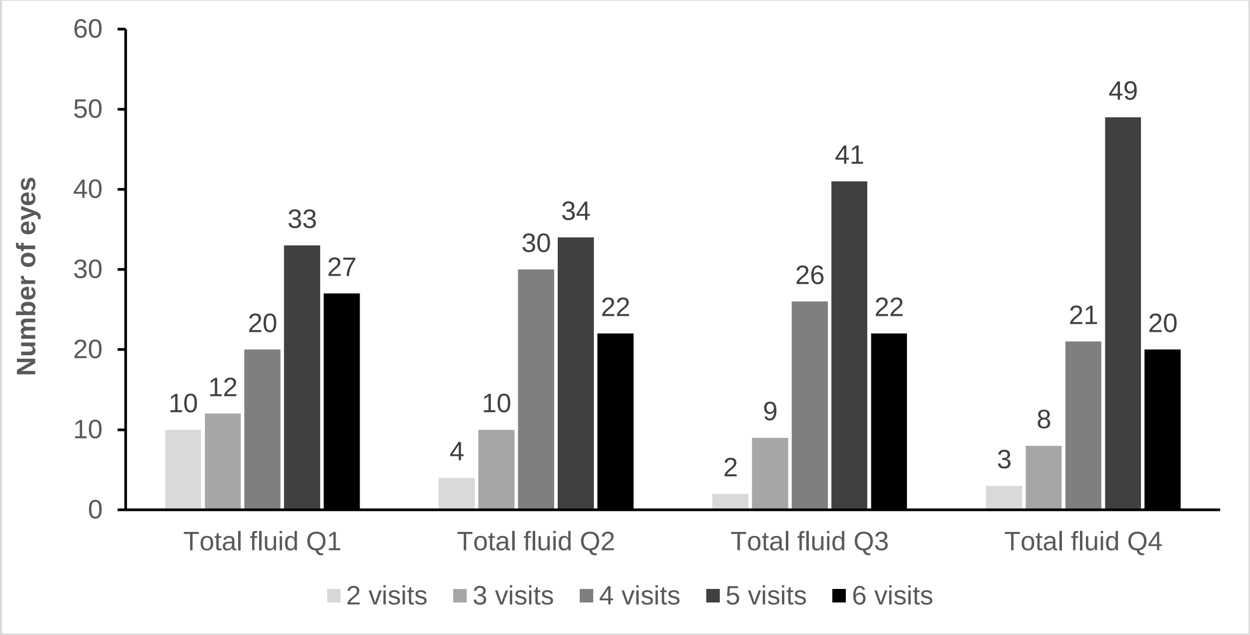

Supplement Figure 3. Correlations between the OCT markers of lesion activity IRF, SRF, PED, CSFT and Total Fluid (IRF+SRF+PED).
Darker shaded cells represent stronger correlations. CSFT, central subfield thickness; IRF, intraretinal fluid; PED, pigment epithelial detachment; SRF, subretinal fluid.

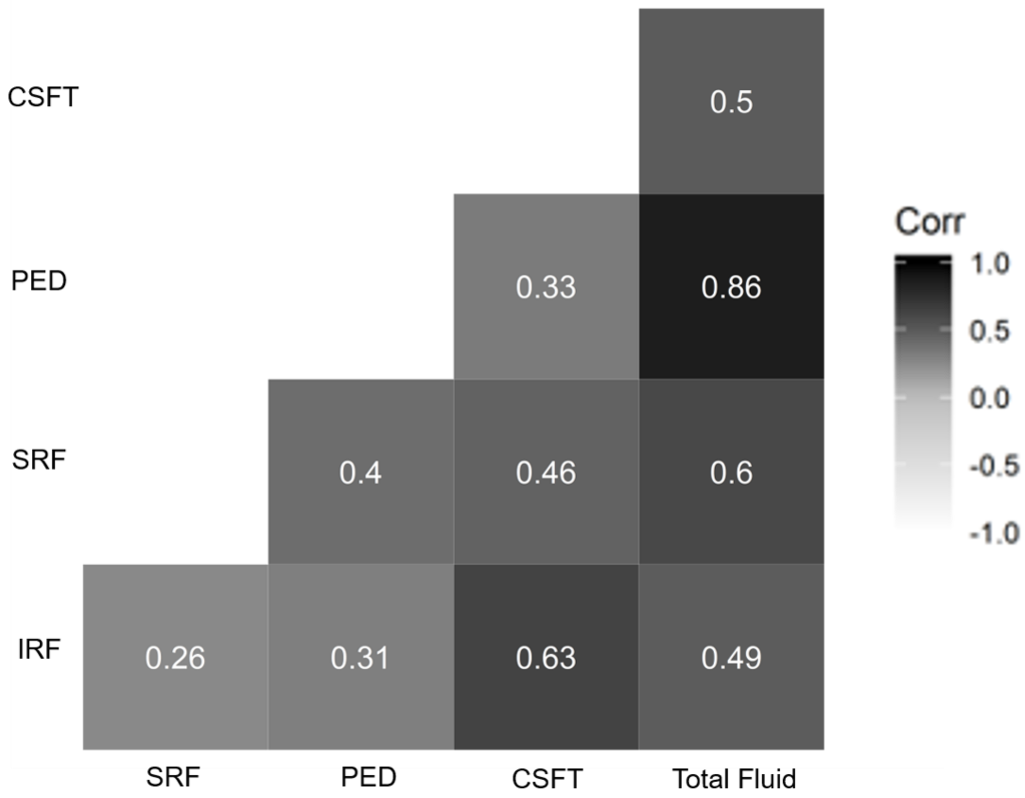

Supplement Figure 4. Mean changes in CSFT over time for eyes in the four CSFT SD quartiles.

CSFT, central subfield thickness; Q, quarter; SD, standard deviation.

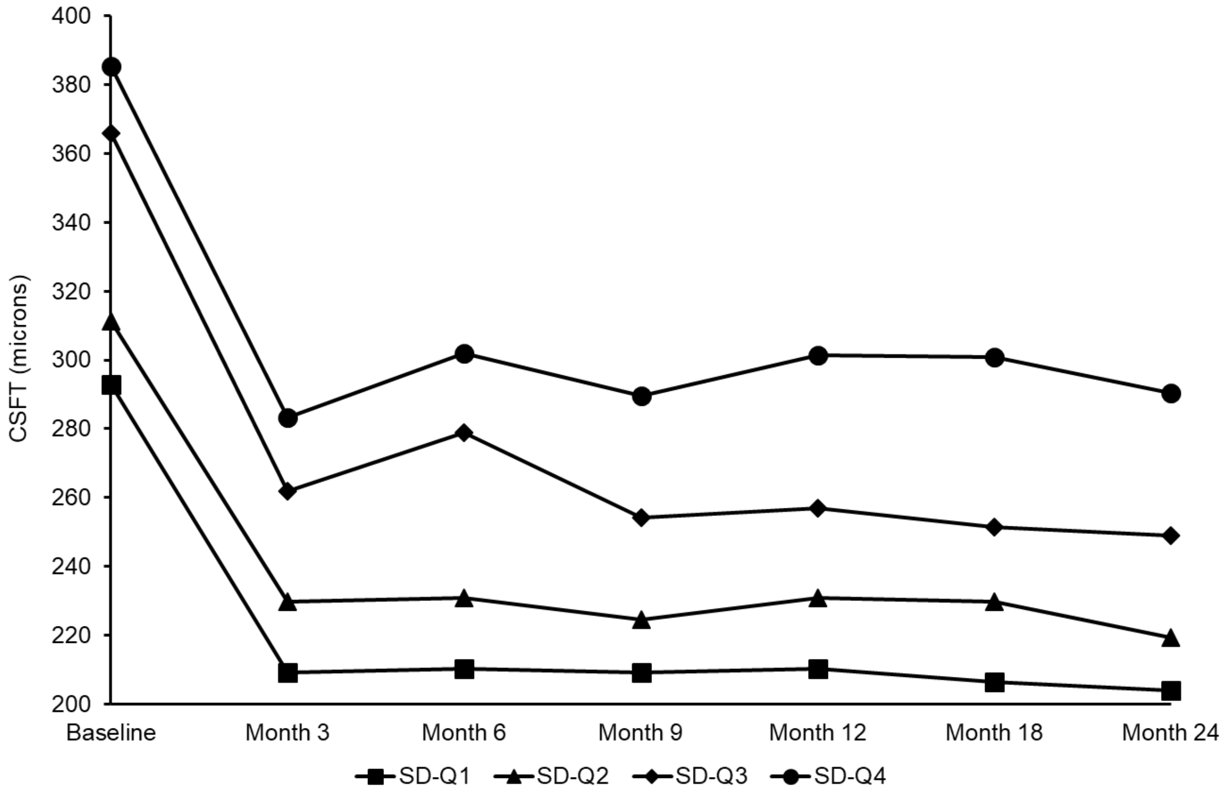

Supplement Figure 5. Trajectory of adjusted estimated marginal mean (LS mean) change in VA from month 3 to month 6, 9, 12, 18 and 24 by SD quartile from GEE models.

(a) IRF, (b) SRF, (c) PED, (d) CSFT, and (e) total fluid (IRF + SRF + PED). The SD across all available visits was calculated for each OCT marker of lesion activity. CSFT, central subfield thickness; GEE, generalized estimating equation; IRF, intraretinal fluid; LS, least squares; OCT, optical coherence tomography; PED; pigment epithelial detachment; SD, standard deviation; SRF, subretinal fluid; VA, visual acuity.

a) IRF

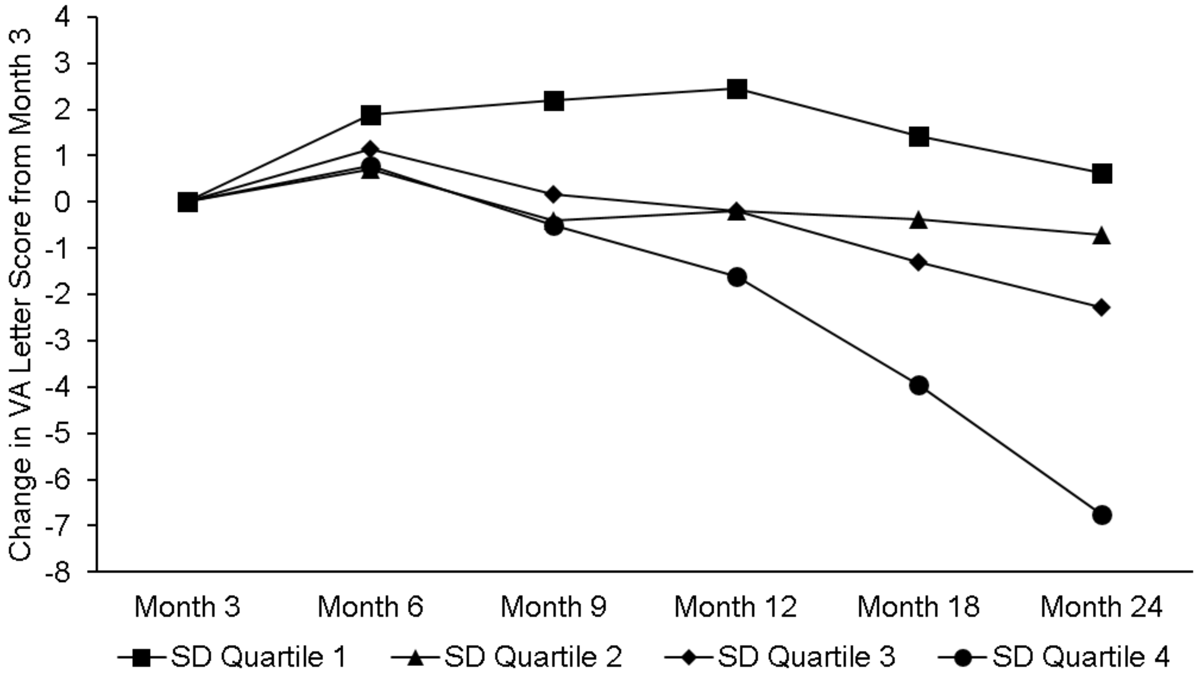

b) SRF

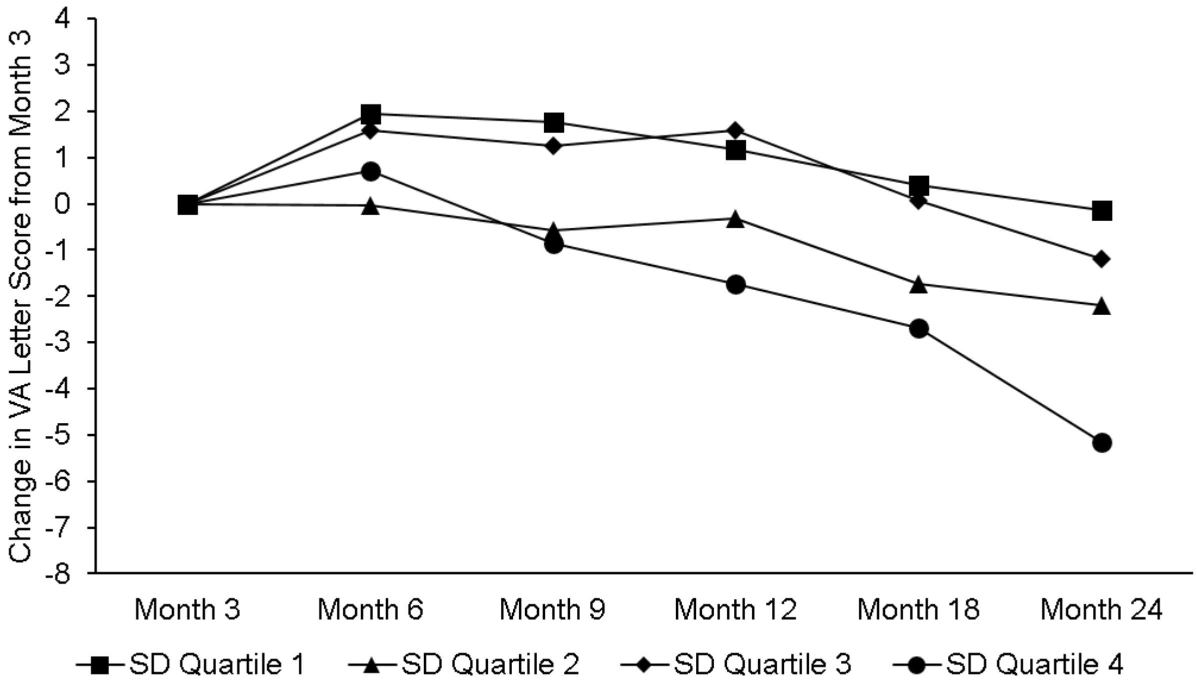
Change in VA Letter Score from Month 3
4
3
2
1
0
-1
-2
-3
-4
-5
-6
-7
-8
Month 3
Month 6
Month 9
Month 12
Month 18
Month 24
SD Quartile 1
SD Quartile 2
SD Quartile 3
SD Quartile 4

c) PED

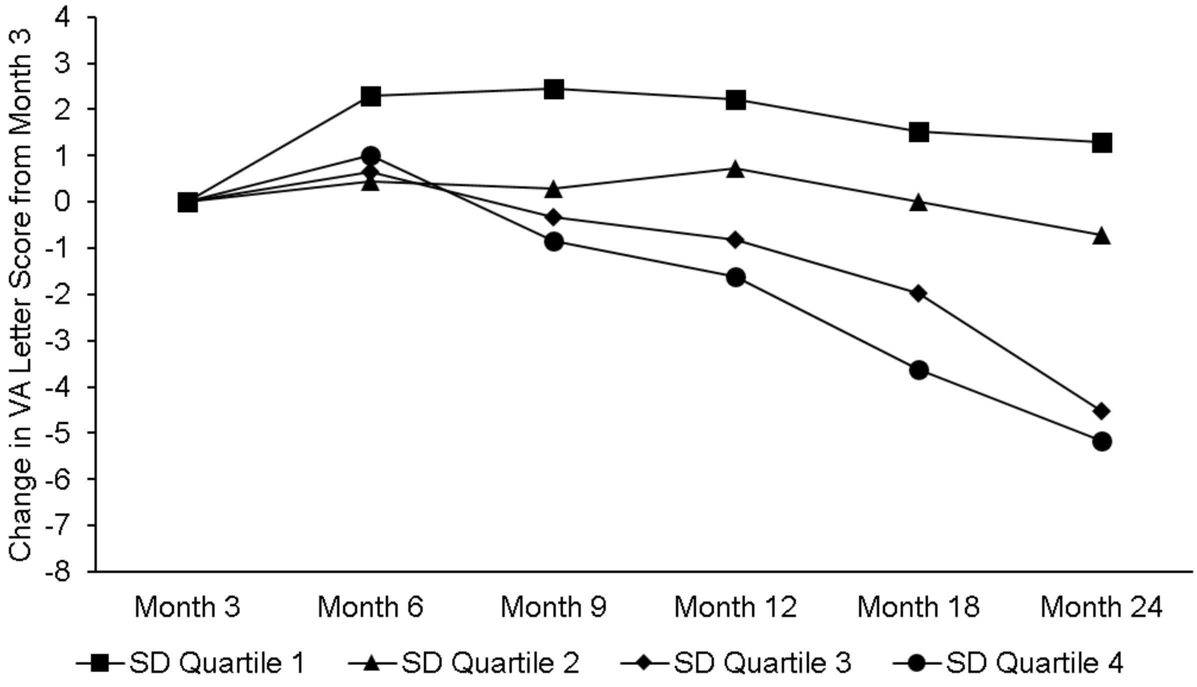
Change in VA Letter Score from Month 3
4
3
2
1
0
-1
-2
-3
-4
-5
-6
-7
-8
Month 3
Month 6
Month 9
Month 12
Month 18
Month 24
SD Quartile 1
SD Quartile 2
SD Quartile 3
SD Quartile 4

d) CSFT

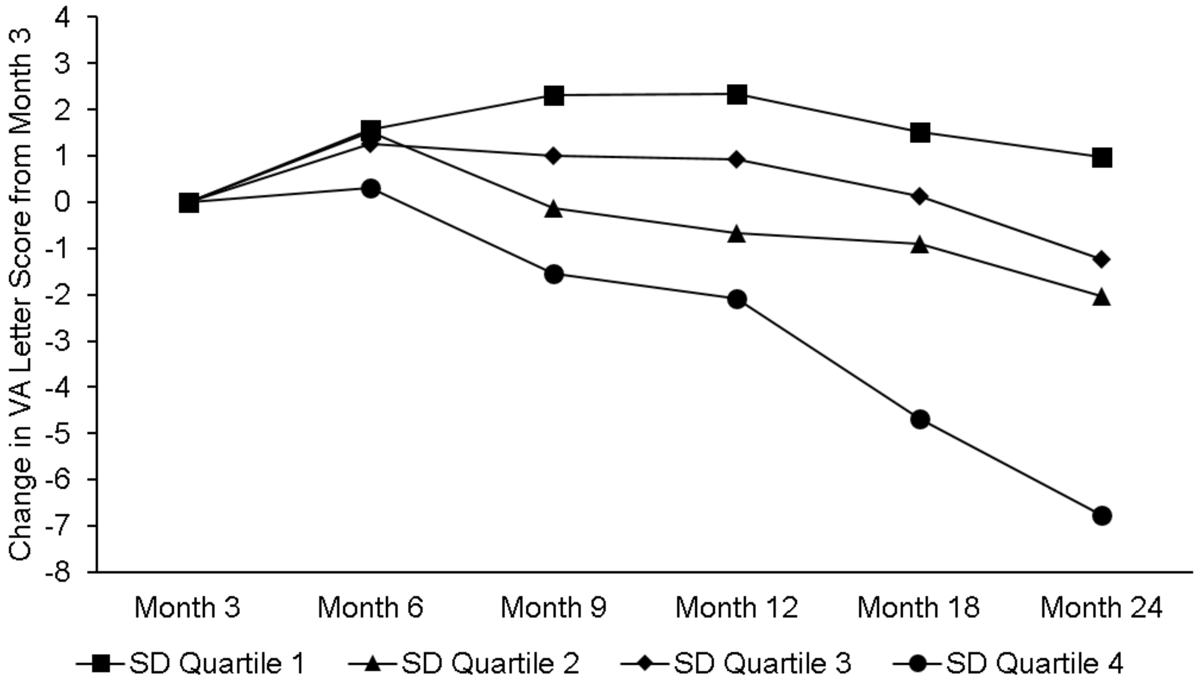

e) Total fluid (IRF+SRF+PED)

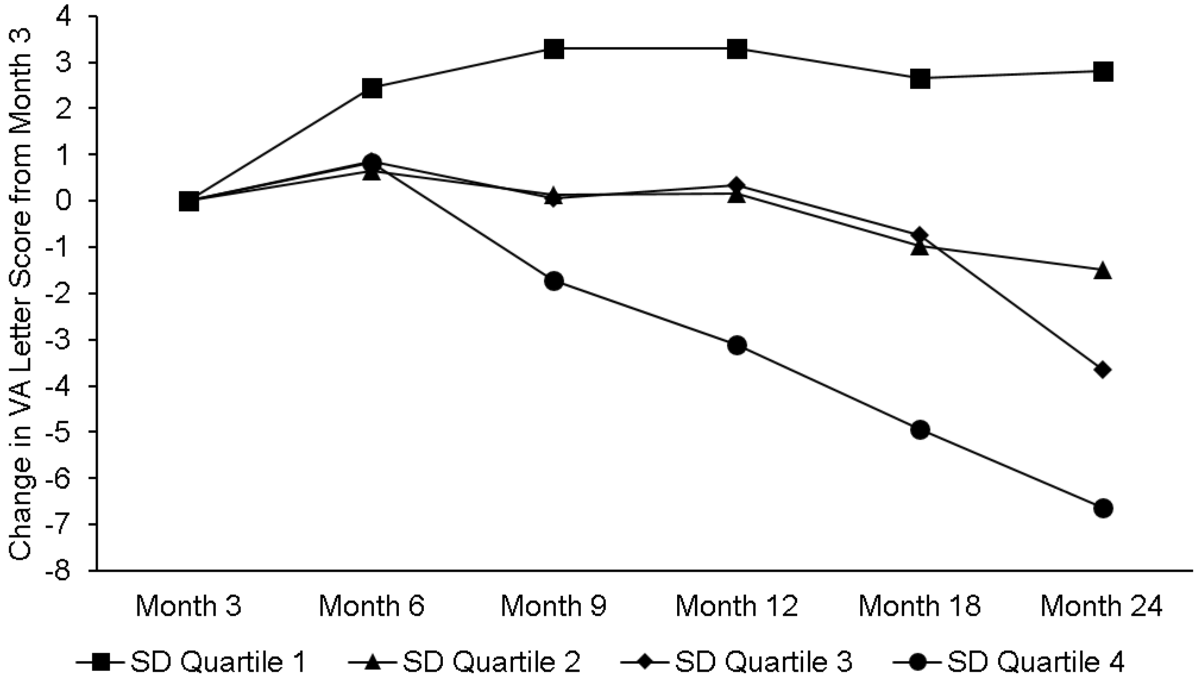

Supplement Figure 6. Box and whisker plots showing distribution of the proportion of clinic visits per eye with presence of IRF and SRF during the maintenance phase of anti-VEGF therapy (Month 3 to Month 24) stratified by SD quartile of CSFT.
The SD across all available visits was calculated for CSFT. CSFT, central subfield thickness; IRF, intraretinal fluid; SD, standard deviation; SRF, subretinal fluid; VEGF, vascular endothelial growth factor.

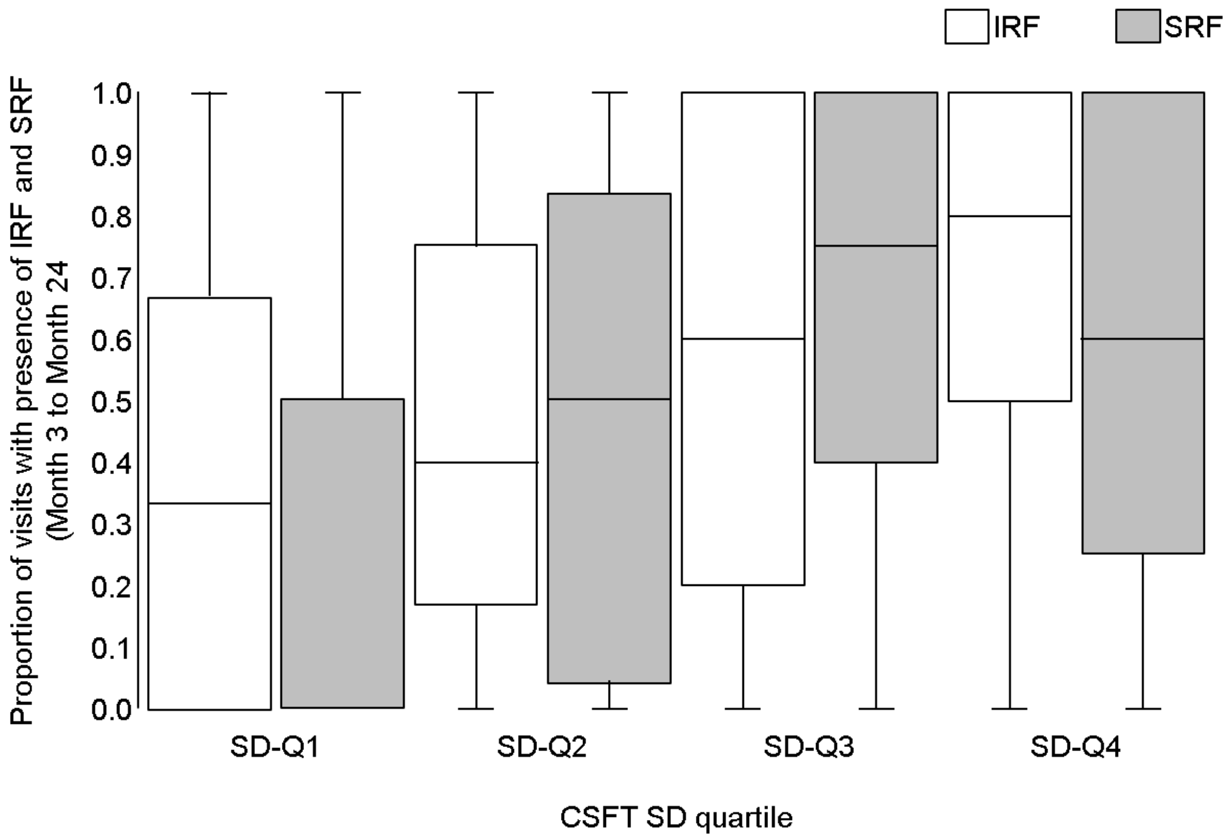

Supplement Table 1. Number of eyes available for analysis in SD quartiles 1 to 4 at Months 3–24.

	**Month 3** (N=393)	**Month 6** (N=361)	**Month 9** (N=361)	**Month 12** (N=386)	**Month 18** (N=398)	**Month 24** (N=403)
**IRF**						
Quartile 1	102	87	92	97	100	102
Quartile 2	95	92	83	94	99	100
Quartile 3	97	90	93	98	100	100
Quartile 4	99	92	93	97	99	101
**SRF**						
Quartile 1	103	94	95	97	104	106
Quartile 2	92	81	86	93	95	95
Quartile 3	99	92	88	96	99	101
Quartile 4	99	94	92	100	100	101
**PED**						
Quartile 1	98	88	88	95	98	101
Quartile 2	98	92	90	96	100	100
Quartile 3	97	87	95	97	101	101
Quartile 4	100	94	88	98	99	101
**CSFT**						
Quartile 1	100	96	88	96	98	101
Quartile 2	95	85	87	95	99	100
Quartile 3	99	91	93	98	100	101
Quartile 4	99	89	93	97	101	101
**Total fluid**						
Quartile 1	99	91	88	97	99	102
Quartile 2	99	85	89	94	100	100
Quartile 3	94	92	95	96	100	100
Quartile 4	101	93	89	99	99	101

CSFT, central subfield thickness; IRF, intraretinal fluid; PED, pigment epithelial detachment; SD, standard deviation; SRF, subretinal fluid.

Supplement Table 2. Cross tabulation of the distribution of eyes in IRF, SRF and PED SD quartiles. Grey shading indicates eyes classified in equivalent SD quartiles (IRF/SRF: 33.2%; IRF/PED: 36.0%; SRF/PED: 39.0%).

		**SRF SD-Quartile**								
		**1**		**2**		**3**		**4**		
		**n**	**%**	**n**	**%**	**n**	**%**	**n**	**%**	
**IRF SD-Quartile**	**1**	43	10.7	17	4.2	24	6.0	18	4.5	
	**2**	31	7.7	27	6.7	24	6.0	18	4.5	
	**3**	22	5.5	23	5.7	27	6.7	28	6.9	**Total**
	**4**	10	2.5	28	6.9	26	6.5	37	9.2	403

		**PED SD-Quartile**								
		**1**		**2**		**3**		**4**		
		**n**	**%**	**n**	**%**	**n**	**%**	**n**	**%**	
**IRF SD-Quartile**	**1**	38	9.4	25	6.2	26	6.5	13	3.2	
	**2**	29	7.2	37	9.2	16	4.0	18	4.5	
	**3**	22	5.5	16	4.0	31	7.7	31	7.7	**Total**
	**4**	12	3.0	22	5.5	28	6.9	39	9.7	403

		**PED SD-Quartile**								
		**1**		**2**		**3**		**4**		
		**n**	**%**	**n**	**%**	**n**	**%**	**n**	**%**	
**SRF SD-Quartile**	**1**	47	11.7	31	7.7	18	4.5	10	2.5	
	**2**	24	6.0	28	6.9	28	6.9	15	3.7	
	**3**	18	4.5	21	5.2	34	8.4	28	6.9	**Total**
	**4**	12	3.0	20	5.0	21	5.2	48	11.9	403

IRF, Intra-retinal fluid; PED, pigment epithelial detachment; SD, standard deviation; SRF, sub-retinal fluid.

Supplement Table 3. Patient and ocular characteristics by IRF SD quartiles in cohorts included in the main and sensitivity analyses.

Features	Limited data cohort (N=413 eyes)	Primary cohort (N=403 eyes from 381 patients)			
		Quartile 1 (Low SD) (N=102 eyes)	Quartile 2 (N=100 eyes)	Quartile 3 (N=100 eyes)	Quartile 4 (High SD) (N=101 eyes)
Age (years; mean [SD])	79.6 (6.3)	78.6 (6.1)	77.2 (7.1)	77.2 (7.0)	78.2 (7.0)
Number of injections during Months 3–24 (mean [SD])	5.4 (4.5)	7.0 (4.8)	8.2 (4.0)	10.2 (2.8)	10.0 (3.3)
Loading phase complete (%)	85.2%	90.1%	88.0%	92.0%	92.1%
VA at baseline (ETDRS letters; mean [SD])	54.6 (11.2)	59.4 (9.7)	60.6 (9.1)	58.0 (10.1)	54.9 (11.9)
VA at Month 3 (ETDRS letters; mean [SD])	57.7 (14.0)	66.2 (11.1)	64.8 (11.3)	63.3 (12.1)	59.0 (14.2)
VA at Month 24 (ETDRS letters; mean [SD])	49.3 (19.4)	66.0 (13.7)	64.4 (13.5)	62.7 (14.8)	53.2 (17.7)
IRF at baseline (mm^3; mean [SD])	0.14 (0.25)	0.05 (0.09)	0.07 (0.18)	0.13 (0.26)	0.31 (0.36)
IRF at Month 3 (mm^3; mean [SD])	0.04 (0.17)	0.00 (0.00)	0.00 (0.00)	0.01 (0.02)	0.06 (0.12)
IRF at Month 24 (mm^3; mean [SD])	0.06 (0.09)	0.00 (0.00)	0.00 (0.00)	0.02 (0.02)	0.11 (0.20)
Change in VA from baseline to Month 3 (ETDRS letters; mean [SD])	3.0 (9.4)	6.8 (9.0)	3.9 (7.9)	5.0 (9.6)	4.0 (11.5)
Change in VA from baseline to Month 24 (ETDRS letters; mean [SD])	-5.2 (18.7)	6.6 (13.1)	3.7 (11.7)	4.8 (14.9)	-1.7 (16.7)
Change in VA from Month 3 to Month 24 (ETDRS letters; mean [SD])	-9.3 (18.1)	-0.2 (10.2)	-0.1 (9.6)	-0.5 (11.7)	-5.6 (13.7)

ETDRS, Early Treatment Diabetic Retinopathy Study; IRF, intraretinal fluid; SD, standard deviation; VA, visual acuity.

Supplement Table 4. Patient and ocular characteristics by SRF SD quartiles in cohorts included in the main and sensitivity analyses.

Features	Limited data cohort (N=413 eyes)	Primary cohort (N=403 eyes from 381 patients)			
		Quartile 1 (Low SD) (N=106 eyes)	Quartile 2 (N=95 eyes)	Quartile 3 (N=101 eyes)	Quartile 4 (High SD) (N=101 eyes)
Age (years; mean [SD])	79.6 (6.3)	80 (6.1)	78.2 (7.9)	76.5 (7)	76.6 (5.7)
Number of injections during Months 3–24 (mean [SD])	5.4 (4.5)	5.9 (4.2)	8.5 (3.5)	10.1 (3.6)	11.0 (2.7)
Loading phase complete (%)	85.2%	84.9%	86.3%	97.0%	94.1%
VA at baseline (ETDRS letters; mean [SD])	54.6 (11.3)	57.7 (10)	58.5 (10.1)	58.1 (10.6)	58.6 (11.1)
VA at Month 3 (ETDRS letters; mean [SD])	57.7 (14.0)	61.7 (13.0)	64.3 (11.5)	63.3 (13.8)	64.2 (11.5)
VA at Month 24 (ETDRS letters; mean [SD])	49.3 (19.4)	60.4 (15.9)	62.8 (15.0)	63.5 (16.4)	59.8 (15.7)
SRF at baseline (mm^3; mean [SD])	0.16 (0.28)	0.10 (0.23)	0.11 (0.18)	0.19 (0.26)	0.31 (0.36)
SRF at Month 3 (mm^3; mean [SD])	0.04 (0.13)	0.00 (0.00)	0.00 (0.00)	0.02 (0.03)	0.10 (0.18)
SRF at Month 24 (mm^3; mean [SD])	0.05 (0.12)	0.00 (0.00)	0.00 (0.00)	0.02 (0.03)	0.13 (0.20)
Change in VA from baseline to Month 3 (ETDRS letters; mean [SD])	3.0 (9.4)	4.0 (9.6)	5.5 (9.2)	5.1 (11.8)	5.4 (7.5)
Change in VA from baseline to Month 24 (ETDRS letters; mean [SD])	-5.2 (18.7)	2.7 (14.2)	4.2 (13.7)	5.4 (15.7)	1.2 (14.2)
Change in VA from Month 3 to Month 24 (ETDRS letters; mean [SD])	-9.3 (18.1)	-1.2 (10.1)	-1.4 (12.0)	0.1 (11.8)	-4.1 (12.3)

ETDRS, Early Treatment Diabetic Retinopathy Study; SD, standard deviation; SRF, subretinal fluid; VA, visual acuity.

Supplement Table 5. Patient and ocular characteristics by PED SD quartiles in cohorts included in the main and sensitivity analyses.

Features	Limited data cohort (N=413 eyes)	Primary cohort (N=403 eyes from 381 patients)			
		Quartile 1 (Low SD) (N=101 eyes)	Quartile 2 (N=100 eyes)	Quartile 3 (N=101 eyes)	Quartile 4 (High SD) (N=101 eyes)
Age (years; mean [SD])	79.6 (6.3)	77.1 (7.8)	77.7 (6.9)	78.8 (6.6)	77.8 (5.8)
Number of injections during Months 3–24 (mean [SD])	5.4 (4.5)	7.4 (4.4)	8.8 (4.1)	9.1 (3.7)	10.1 (3.3)
Loading phase complete (%)	85.2%	84.2%	91.0%	92.1%	95.0%
VA at baseline (ETDRS letters; mean [SD])	54.6 (11.3)	58.9 (10.5)	57.9 (10.0)	57.8 (10.6)	58.4 (10.7)
VA at Month 3 (ETDRS letters; mean [SD])	57.7 (14.0)	64.6 (11.7)	63.5 (11.8)	62.8 (14.6)	62.5 (11.8)
VA at Month 24 (ETDRS letters; mean [SD])	49.3 (19.4)	65.4 (14.6)	62.5 (15.0)	60.1 (16.1)	58.4 (16.7)
PED at baseline (mm^3; mean [SD])	0.74 (0.82)	0.35 (0.31)	0.46 (0.37)	0.75 (1.16)	1.36 (1.23)
PED at Month 3 (mm^3; mean [SD])	0.59 (0.64)	0.26 (0.27)	0.36 (0.29)	0.45 (0.30)	0.91 (0.68)
PED at Month 24 (mm^3; mean [SD])	1.07 (1.01)	0.28 (0.27)	0.41 (0.29)	0.53 (0.30)	0.96 (0.68)
Change in VA from baseline to Month 3 (ETDRS letters; mean [SD])	3.0 (9.4)	5.4 (9.1)	5.4 (9.2)	5.1 (11.8)	4.0 (8.0)
Change in VA from baseline to Month 24 (ETDRS letters; mean [SD])	-5.2 (18.7)	6.5 (13.1)	4.6 (12.5)	2.3 (16.1)	0.0 (15.4)
Change in VA from Month 3 to Month 24 (ETDRS letters; mean [SD])	-9.3 (18.1)	1.2 (10.0)	-0.5 (9.9)	-3.0 (11.8)	-4.2 (13.7)

ETDRS, Early Treatment Diabetic Retinopathy Study; PED, pigment epithelial detachment; SD, standard deviation; VA, visual acuity.

Supplement Table 6. Patient and ocular characteristics by CSFT SD quartiles in cohorts included in the main and sensitivity analyses.

Features	Limited data cohort (N=413 eyes)	Primary cohort (N=403 eyes from 381 patients)			
		Quartile 1 (Low SD) (N=102 eyes)	Quartile 2 (N=100 eyes)	Quartile 3 (N=100 eyes)	Quartile 4 (High SD) (N=101 eyes)
Age (years; mean [SD])	79.6 (6.3)	78.4 (6.9)	78.6 (6.1)	77.0 (7.0)	77.4 (7.2)
Number of injections during Months 3–24 (mean [SD])	5.4 (4.5)	6.1 (4.6)	9.0 (3.6)	10.3 (3.2)	10 (3.2)
Loading phase complete (%)	85.2%	88.1%	89.0%	97.0%	88.1%
VA at baseline (ETDRS letters; mean [SD])	54.6 (11.3)	59.0 (10.1)	59.5 (9.8)	57.8 (10.7)	56.8 (10.9)
VA at Month 3 (ETDRS letters; mean [SD])	57.7 (14.0)	64.0 (12.6)	64.4 (12.5)	63.4 (11.7)	61.6 (13.2)
VA at Month 24 (ETDRS letters; mean [SD])	49.3 (19.4)	64.5 (15.7)	62.5 (13.9)	63.8 (14.4)	55.7 (17.5)
CSFT at baseline (microns; mean [SD])	337.1 (109.3)	322.8 (109.1)	325.6 (81.3)	338.7 (109.0)	411.7 (112.9)
CSFT at Month 3 (microns; mean [SD])	240.5 (71.4)	216.9 (36.6)	228.1 (34.9)	260.5 (60.3)	286.8 (93.2)
CSFT at Month 24 (microns; mean [SD])	257.7 (64.0)	212.5 (44.2)	225.1 (41.3)	250.1 (56.3)	269.2 (90.3)
Change in VA from baseline to Month 3 (ETDRS letters; mean [SD])	3.0 (9.4)	4.9 (7.4)	4.8 (11.0)	5.4 (8.0)	4.7 (11.6)
Change in VA from baseline to Month 24 (ETDRS letters; mean [SD])	-5.2 (18.7)	5.5 (12.3)	3.0 (13.4)	6.0 (14.2)	-1.1 (16.9)
Change in VA from Month 3 to Month 24 (ETDRS letters; mean [SD])	-9.3 (18.1)	0.4 (10.3)	-1.7 (10.2)	0.5 (11.3)	-5.8 (13.3)

CSFT, central subfield thickness; ETDRS, Early Treatment Diabetic Retinopathy Study; SD, standard deviation; VA, visual acuity.

Supplement Table 7. Patient and ocular characteristics by total fluid (IRF+SRF+ PED) SD quartiles in cohorts included in the main and sensitivity analyses.

Features	Limited data cohort (N=413 eyes)	Primary cohort (N=403 eyes from 381 patients)			
		Quartile 1 (Low SD) (N=102 eyes)	Quartile 2 (N=100 eyes)	Quartile 3 (N=100 eyes)	Quartile 4 (High SD) (N=101 eyes)
Age (years; mean [SD])	79.6 (6.3)	77.9 (7.6)	77.6 (7.1)	78.4 (6.6)	77.4 (6.0)
Number of injections during Months 3–24 (mean [SD])	5.4 (4.5)	6.9 (4.3)	8.6 (4.3)	9.4 (3.4)	10.5 (3.1)
Loading phase complete (%)	85.2%	84.3%	93.0%	89.0%	96.0%
VA at baseline (ETDRS letters; mean [SD])	54.6 (11.3)	59.2 (10.2)	58.1 (10.0)	58.8 (10.3)	56.8 (11.2)
VA at Month 3 (ETDRS letters; mean [SD])	57.7 (14.0)	63.7 (13.0)	63.7 (13.2)	65.5 (11.4)	60.7 (11.9)
VA at Month 24 (ETDRS letters; mean [SD])	49.3 (19.4)	65.6 (15.2)	62.7 (15.1)	62.2 (15.1)	55.8 (16.3)
Total fluid volume at baseline (mm^3; mean [SD])	1.04 (0.99)	0.55 (0.41)	0.82 (1.29)	0.95 (0.64)	1.84 (1.54)
Total fluid volume at Month 3 (mm^3; mean [SD])	0.68 (0.76)	0.26 (0.22)	0.42 (0.34)	0.56 (0.41)	0.96 (0.76)
Total fluid volume at Month 24 (mm^3; mean [SD])	1.17 (1.07)	0.29 (0.2)	0.47 (0.33)	0.63 (0.38)	1.08 (0.76)
Change in VA from baseline to Month 3 (ETDRS letters; mean [SD])	3.0 (9.4)	4.3 (10.9)	5.6 (10.1)	6.2 (8.6)	3.9 (8.6)
Change in VA from baseline to Month 24 [ETDRS letters]	-5.2 (18.7)	6.4 (13.8)	4.6 (13.8)	3.4 (14.2)	-1.1 (15.4)
Change in VA from Month 3 to Month 24 (ETDRS letters; mean [SD])	-9.3 (18.1)	2.2 (10.0)	-0.7 (9.4)	-3.1 (12.3)	-5.0 (13.2)

ETDRS, Early Treatment Diabetic Retinopathy Study; IRF, intraretinal fluid; PED, pigment epithelial detachment; SD, standard deviation; SRF, subretinal fluid; VA, visual acuity.

Supplement Table 8. Number of injections and changes in VA over time in eyes without missing OCT metrics (N=403) compared with eyes with missing metrics (N=413).

Characteristics	Primary cohort (N=403 eyes from 381 patients)	Limited data cohort (N=413 eyes from 399 patients)
Loading phase complete (%)	90.6%	85.2%
Number of injections during Months 3–24 (mean [SD])	8.9 (4.0)	5.4 (4.5)
Annual injection rate (mean [SD])	4.6 (2.1)	3.1 (2.6)
Change in VA from baseline to Month 3 (ETDRS letters; mean [SD])	5.0 (9.6)	3.0 (9.4)
Change in VA from baseline to Month 24 (ETDRS letters; mean [SD])	3.4 (14.5)	-5.2 (18.7)*
Change in VA (imputed when missing) from baseline to Month 24 (ETDRS letters; mean [SD])	NA	-4.2 (15.6)**
Change in VA from Month 3 to Month 24 (ETDRS letters; mean [SD])	-1.6 (11.6)	-9.3 (18.1)*
Change in VA (imputed when missing) from Month 3 to Month 24 (ETDRS letters; mean [SD])	NA	-7.0 (13.2)

*n=185 eyes with VA data at Month 24; **For the 228 eyes with missing VA information at Month 24, a single mean imputation model was applied, including the covariates age, baseline VA, loading phase complete (yes/no), VA trend prior to Month 24 (difference between the two most recent VA measurement prior to Month 24), most recent VA measurement prior to Month 24. ETDRS, Early Treatment Diabetic Retinopathy Study; NA, not applicable; OCT, optical coherence tomography; SD, standard deviation; VA, visual acuity.
